# Errors in Radiology: A Standard Review

**DOI:** 10.3390/jcm13154306

**Published:** 2024-07-23

**Authors:** Filippo Pesapane, Giulia Gnocchi, Cettina Quarrella, Adriana Sorce, Luca Nicosia, Luciano Mariano, Anna Carla Bozzini, Irene Marinucci, Francesca Priolo, Francesca Abbate, Gianpaolo Carrafiello, Enrico Cassano

**Affiliations:** 1 Breast Imaging Division, Radiology Department, IEO European Institute of Oncology IRCCS, Via Giuseppe Ripamonti 435, 20141 Milan, Italy; luca.nicosia@ieo.it (L.N.); luciano.mariano@ieo.it (L.M.); anna.bozzini@ieo.it (A.C.B.); irene.marinucci@ieo.it (I.M.); francesca.priolo@ieo.it (F.P.); francesca.abbate@ieo.it (F.A.); enrico.cassano@ieo.it (E.C.); 2 Postgraduation School in Radiodiagnostics, Università degli Studi di Milano, Via Festa del Perdono, 7, 20122 Milan, Italy; giulia.gnocchi@unimi.it (G.G.); cettina.quarrella@unimi.it (C.Q.); adriana.sorce@unimi.it (A.S.); gianpaolo.carrafiello@unimi.it (G.C.); 3 Radiology Department, Fondazione IRCCS Cà Granda, Policlinico di Milano Ospedale Maggiore, Università di Milano, 20122 Milan, Italy

**Keywords:** diagnostic, errors, radiology, methodology, AI

## Abstract

Radiological interpretations, while essential, are not infallible and are best understood as expert opinions formed through the evaluation of available evidence. Acknowledging the inherent possibility of error is crucial, as it frames the discussion on improving diagnostic accuracy and patient care. A comprehensive review of error classifications highlights the complexity of diagnostic errors, drawing on recent frameworks to categorize them into perceptual and cognitive errors, among others. This classification underpins an analysis of specific error types, their prevalence, and implications for clinical practice. Additionally, we address the psychological impact of radiological practice, including the effects of mental health and burnout on diagnostic accuracy. The potential of artificial intelligence (AI) in mitigating errors is discussed, alongside ethical and regulatory considerations in its application. This research contributes to the body of knowledge on radiological errors, offering insights into preventive strategies and the integration of AI to enhance diagnostic practices. It underscores the importance of a nuanced understanding of errors in radiology, aiming to foster improvements in patient care and radiological accuracy.

## 1. Introduction

Radiologists play a critical role in interpreting medical images, providing expert opinions that significantly impact patient care. These interpretations are not absolute truths but rather informed opinions generated through a meticulous evaluation of the available evidence. This understanding acknowledges the inherent limitations of radiological interpretations, which, while invaluable, cannot supplant the definitive diagnoses provided by histological or microbiological examinations.

The radiologist’s job is to interpret images and answer specific clinical questions. This interpretation should not be considered incontrovertible; radiology aims to provide a presumptive diagnosis, not to substitute histological or microbiological diagnoses [1]. Radiologists offer expert opinions, where the term “opinion,” according to the Longman Dictionary of English Language (1984), refers to a conclusion reached after weighing evidence but open to debate and suggestions [2].

Several factors influence these interpretations: the quality and appropriateness of the images, the radiologist’s knowledge, availability of previous imaging for comparison, patient’s clinical history, and direction from physicians on the most probable diagnostic hypotheses. To classify an interpretation as an error, the radiologist must have had all the necessary tools to make a correct diagnosis and still miss it without any possibility for disagreement [3].

Recognizing potential causes of error in clinical practice is crucial for finding solutions to minimize them. Accordingly, this paper seeks to explore the multifaceted dimensions of radiological interpretation, differentiating between the subjective nature of radiological opinions and the objective identification of errors. In doing so, it underscores the importance of acknowledging the potential for error inherent in the practice of radiology and the consequent need for a robust framework to classify and address these errors. This exploration is grounded in the recognition that radiological interpretations are influenced by a constellation of factors, including the quality and relevance of the images, the radiologist’s expertise, and the integration of the patient’s clinical history and previous imaging studies. This paper further delves into the classification of errors, drawing on contemporary frameworks that offer a comprehensive categorization of error types, thereby facilitating a deeper understanding of the sources and nature of errors in radiological practice.

Moreover, this paper examines the dichotomy between person-centered and system approaches to understanding human error in radiology, advocating for a more holistic view that considers the systemic factors contributing to errors. This perspective is crucial for developing strategies that enhance the accuracy of radiological interpretations and minimize the occurrence of errors.

This classification serves as a foundation for discussing specific error types, including perceptual and cognitive errors, and the strategies employed to mitigate these errors, such as continuous education and the utilization of artificial intelligence (AI).

Furthermore, this paper explores the psychological and systemic factors that influence radiological interpretations, including the impact of mental health and burnout on radiologists’ performance. It acknowledges the challenging nature of radiological work, characterized by high demands and the critical need for accuracy, and the toll this can take on radiologists’ well-being.

This review aims to contribute to the ongoing dialog on enhancing the accuracy and reliability of radiological practices, ultimately improving patient care.

## 2. Person-Centered vs. System Approach

Human error can be seen with a person-centered approach or a system approach [4]. The first one focuses on the individual who makes the mistake and relies on psychological factors such as carelessness, forgetfulness, poor motivation, and inattention. The second one assumes that error is the result of multiple factors; because humans are fallible by definition and prone to making mistakes, the best way to reduce errors is to improve the environment and the conditions in which physicians operate [5].

## 3. Classification of Errors

Over time, various classifications have been proposed to typify errors, including the one by Brook et al. [6], Pinto and Brunese [7], and Provenzale and Kranz [8]. The most recent and used classification to categorize the different types of error is the one by Kim and Mansfield [9], which is a revision of the previous classification by Renfrew [10]. Two radiologists reviewed and classified 656 radiologic examinations with delayed diagnoses, collected from 1 July 2002 to 31 January 2010. Among them, a total of 1269 errors were found. Each diagnostic error was then included into twelve main categories, five more than the ones by Renfrew. This is obviously a simplification since errors may be due to a combination of these factors. 

The imaging study most prone to error is radiography, particularly in the musculoskeletal area [9].

Type 4 error (underreading) is the most common one (42%), followed by type 10 (satisfaction of search) (22%) and type 2 (faulty reasoning) errors (9%) [9].

Complacency (type 1 error) occurs when a finding is appreciated but it is attributed to the wrong cause [9]. This scenario may lead to a false positive error and may consequently cause unnecessary diagnostic and therapeutic effort [11].

Faulty reasoning (type 2 error) occurs when a finding is appreciated and interpreted as abnormal but is attributed to the wrong cause. This type of error is particularly prone to cognitive bias, whether from misleading clinical information or an overly limited differential diagnosis [12].

Type 3 error is the wrong interpretation of an abnormal radiological finding correctly identified on the imaging study because of the lack of knowledge of the radiologist who analyzed it [13]. Over time, to reduce this type of error, there was an increasing tendency of radiologists to make descriptive reports rather than concrete diagnostic hypotheses. This may be due to fear of repercussions if an uncurrent diagnosis is formulated. However, trying to give a first and last name to an abnormality should be the goal of each radiological report. The European Society of Radiology itself published a handbook that highlights the importance of adding a conclusion in making a report, keeping in mind that, in some cases, it is the only section read by physicians [14,15].

Continuous training through attending courses, studying the literature, and conducting research can lead to greater knowledge and reduce this type of error. Faced with a doubtful finding, a radiologist should have the humility to seek advice from more experienced colleagues and, at the same time, transmit their knowledge to others when they learn something new.

Underreading (type 4 error) means that a finding is visible on the imaging study but missed by the radiologist. This error is also the most common in the insurance claims for criminal or civil liability in radiology (66.7%) [16]. One of the most recognized tools to overcome it is the use of checklists, which guide the radiologist in the diagnosis, and which may be customized according to the body district studied [17,18].

Even an experienced radiologist can miss evident radiological signs of disease. In assessing the legal implications of this error, one cannot ignore so-called hindsight [19]. To understand the concept, just think of Escher’s paradoxical drawings or the children’s book “Where is Wally?”, in which children are asked to find Wally in drawing scenarios with a large number of similar-looking people [20]. If finding him may seem difficult in the beginning, once you discover his location—by yourself or by looking at solutions—his presence on the pages appears obvious and easy to recognize [13].

Some medical errors can be the result of poor communication (type 5) between radiologists, patients, and other clinicians. Communication of radiological findings must be effective in order to provide appropriate care for patients. Especially when findings are significant or unexpected, radiologists should directly communicate with referring physicians to take immediate actions. For these reasons, the European Society of Radiology has published communication guidelines for radiologists to provide helpful information on how to conduct an effective discussion between patients, referrers, colleagues, and students [21].

Furthermore, simulation scenarios can help improve communication and teamwork, thus decreasing errors and improving team morale [22].

Type 6 error is ascribable to the technique. It occurs when a finding is missed because of the limitations of the examination or technique [12]. In these cases, “follow-up or additional diagnostic studies to clarify or confirm the impression should be suggested when appropriate”, according to The American College of Radiology (ACR) Practice Guideline for Communication of Diagnostic Imaging Findings [23].

Type 7, or prior examination error, results from neglecting to review the patient’s previous radiologic studies or reports. Acquiring knowledge of the patient’s clinical and radiological history not only enables new lesions’ identification but also facilitates the study of the progression over time of a pre-existing pathology, and its potential response to treatment. For example, it was demonstrated that conducting comparisons with previous mammograms markedly enhances overall performance and can minimize unnecessary referrals attributed to non-lesion sites [24].

A lack of knowledge about the patients’ medical history can lead to an incorrect interpretation of the images (type 8 error). For example, radiotherapy for chest wall or intrathoracic malignancies may be the cause of pneumonitis and fibrosis [25], and the correct evaluation of the chest CT scan of a patient undergoing this type of treatment would only be possible by knowing the disease and the oncological therapy to which they are undergoing. To reduce this type of error, better interprofessional communication between clinicians and radiologists must be incentivized. However, clinical information may lead to false positive reports. To avoid being overly influenced by them, the radiologist should look at the images prior to reading the patient’s medical history and their clinical data [26].

Type 9 error is location. This is the failure to recognize a pathological finding visible within the confines of the examination, yet lying beyond the intentionally scrutinized region of interest, usually at the edges of the assessed area [3]. To overcome type 9 error, enhanced training and education are crucial, emphasizing the importance of evaluating the entire image, including peripheral areas. Advanced imaging techniques, such as wider-field-of-view imaging and multiplanar reconstruction, help ensure comprehensive coverage. CAD systems, particularly those incorporating AI and machine learning, can automatically analyze images and highlight potential abnormalities that might be overlooked. Moreover, structured reporting with checklists ensures systematic review of all image regions, and double reading by another radiologist can provide an additional layer of scrutiny.

Satisfaction of search error (type 10) is made when a clinically significant abnormality is missed after a first less-important finding is identified. The concept can be explained by referring to the phenomenon of inattentional blindness studied in 1999 by Simons and Chabris [27]. Their conclusion was that humans perceive and remember only things that receive focused attention. A similar experiment has also been carried out in the radiological field. Twenty-four radiologists were asked to perform a familiar lung nodule detection task. In the last CT scan, a gorilla 48 times larger than the average nodule was inserted. Eighty-three percent of radiologists failed to recognize it [28].

Type 11 error is complications, including adverse events occurring during or after radiological procedures. Complications include contrast agent extravasation, allergic reactions, nephrotoxicity, and interventional radiology (IR) procedures. Unlike diagnostic radiology, IR has unique considerations as to how error can occur, such as functional equipment, available technology, or information provided by others, resulting in an incomplete clinical picture [3].

Acknowledging the unique factors that facilitate adverse events in IR and continuing the development of safety practices may reduce the risk of medical error and patient harm [29].

Satisfaction of report (type 12) occurs when the radiologist relies too much on the reports of the patient’s previous examinations. Also called alliterative error, it is the influence that a radiologist can have on another [30]. As a result, if a first radiologist made a wrong assessment in their report, a second radiologist will be more prone to making the same mistake in their subsequent evaluation. This does not mean that comparison with previous exams should be avoided, as it is able to improve diagnostic accuracy [31]. To overcome this error, the radiologist should only read old reports after having independently evaluated the images and drawn their own conclusions.

## 4. Cause of Errors

### 4.1. Perceptual vs. Cognitive Errors

Diagnostic errors can also be divided into perceptual errors and cognitive errors.

Perceptual errors, the most common type, take place when an important abnormality is not identified on the images. They are related to specific risk factors, such as conspicuity of the target lesion on the image, reader fatigue, an overly rapid pace of performing interpretations, satisfaction of search, distractions, or interruptions [12]. AI can increase the detection of potential abnormalities, such as tumors, recognizing changes in intensity, or the appearance of unusual patterns.

Common perceptual errors are, for example, missed lung nodules. Detecting lung nodules is important to diagnose early lung cancer and consequently improve the prognosis of patients.

The reasons for misdiagnosis on CT scans can be related to specific characteristics of the undetected lesion, such as small size, ground-glass appearance, and central location [32].

Missed fractures can be common perceptual errors too. In a retrospective study by Guernazi et al. [33], AI-assisted radiographic readings by six types of readers showed a 10.4% improvement in fracture detection sensitivity without specificity reduction. AI assistance shortened the radiograph reading time by 6.3 s per patient and significantly improved the sensitivity detection of fracture in all locations but shoulder, clavicle, and thoracolumbar spine.

Cognitive errors occur when the abnormality is visually detected but its meaning is not correctly understood. This type of error may be due to a lack of knowledge, a cognitive bias on the part of the radiologist interpreting the study, misleading clinical information, or an error made by a colleague in a previous radiology report [12].

Many common cognitive errors are often secondary to poor knowledge of findings and differential diagnosis.

Thus, after the identification of a suspicious lesion, AI could help with its characterization, defining the boundary extent and differentiating between benign vs malignant.

A prospective, multicenter study by Wei et al. explored the diagnostic value of computer-aided diagnosis (CAD) software (S-Detect, Samsung Ultrasound RS80A software, Samsung Medison Co. Ltd., Seoul, Republic of Korea) on ultrasound in distinguishing benign and malignant breast masses. It demonstrated that CAD software is a non-invasive technique with a high diagnostic value that may be used as an effective auxiliary diagnostic tool for the differential diagnosis of breast massed and reducing unnecessary biopsy [34].

### 4.2. Mental Health and Burnout

Radiologists are encouraged to do more with less. Radiologists are constantly challenged to work more hours, report more images, cover more diagnostics, but, at the same time, care for more patients, as well as maximize the result by making as few mistakes as possible.

Fatigue, both physical and mental, is often an underestimated source of error. 

Krupinski et al. have dedicated part of their careers to analyzing the role of fatigue in radiologists’ perception during their work [35]. They demonstrated that the ability to detect fractures and focus on skeletal radiographs significantly decreased after a long working day [36]. The same happened in the detection of lung nodules on CT scans [37].

Ruutiainen et al. observed that the major discrepancies between resident preliminary reports and faculty final reports increased particularly during the last two hours of consecutive 12 h overnight call shifts, with 29% of all errors occurring during that final block of time [38].

Different tests were proposed to measure perceived fatigue in work environments. The Swedish Occupational Fatigue Inventory (SOFI) is one of them and is based on five different topics: lack of energy, physical exertion, physical discomfort, lack of motivation, and sleepiness [39].

Mental health is a delicate and underestimated issue. It seems that society often forgets that radiologists, like any other health professionals, are first of all people. And that the responsibility of their role, dealing with life and death, is something huge. This issue has been certainly accentuated by the COVID-19 pandemic, in which healthcare professionals have found themselves fighting an invisible enemy with few weapons at their disposal, working until they experience burnout [40,41]. In 2020, a 43-item anonymous questionnaire was submitted to radiologists all over the United States [42]. Over half (61%) of them rated their anxiety levels to be seven or higher. Physician burnout is not only associated with increased rates of depression, anxiety, alcohol and drug abuse, and difficult relationships with coworkers but may also result in increased medical errors [43].

### 4.3. Visual Fatigue

Radiologists spend prolonged time behind computer displays, and visual fatigue must also be considered as a potential source of errors. Prolonged hours of digital screen exposure may produce symptoms such as eye strain, headache, and asthenopia, a pattern that is included under the name of computer vision syndrome (CVS) [44].

Recommendations to reduce visual fatigue include the use of blue light filtering glasses [45], taking frequent microbreaks from the computer terminal [46], and following the “20–20–20” rule: for every 20 min a person looks at a screen, they should shift their eyes to look at something 20 feet away for at least 20 s. The computer display should also be tilted slightly down to reduce screen glare, and the digital screen brightness should not exceed the one of the surrounding environments [46].

## 5. Variability in Error Rates Worldwide

The analysis of the diagnostic error rate in radiology cannot overlook the differences in healthcare systems. These differences could stem from various factors, including the availability of resources, implemented healthcare policies, and disparities in quality control procedures.

For instance, the introduction of second reading policies, where a second radiologist independently reviews the images, can vary and impact the identification of errors.

Exploring how the variability in error rates differs among different regions and healthcare systems is crucial for identifying best practices and implementing targeted improvements.

In a study conducted in Canada by Chan et al. [47], it was concluded that implementing a double reading of screening breast MRI scans significantly reduces the number of unnecessary biopsies. Similarly, the experience of Brown et al. in a United Kingdom hospital has shown that the double reading of screening mammograms detects more cancers compared to single reading [48].

Countries with limited resources may encounter a higher rate of errors due to the absence of advanced diagnostic technologies (type 6 error) [23,49].

Finally, differences in the training of radiologists among countries can influence the rate of diagnostic errors [50].

A recent study conducted in Spain by Cardenas et al. identified the 10 neuroradiological emergencies considered most challenging by radiology residents [51]. A total of 90% of residents declared fear of early cerebral edema due to the subtle nature of initial findings and concerns about false-positive errors. They also cited unfamiliarity with the normal appearance of cerebral spinal fluid spaces in children. Meanwhile, 73% of residents were apprehensive about detecting dural sinus thrombosis in non-contrast head CT scans, considering its relative rarity and that it is difficult to suspect clinically. The differential diagnosis between retropharyngeal edema and the early stage of an abscess was also challenging for 63% of trainees. In such cases, it is advisable to prioritize caution, carefully consider the patient’s medical history, and not solely concentrate on changes in mental status [51].

A study conducted in a trauma center in Israel showed that the most common CT-missed diagnoses among radiology residents were chronic infarctions, hypodense lesions, and mucosal thickening of the paranasal sinuses [52].

## 6. Technological Advances for Reducing Errors in Radiology

In the current era of artificial intelligence (AI), its application in clinical imaging may offer support in reducing the error rate of radiologists.

Prolonged screen exposure, particularly during the search for small lung nodules, seems to be associated with a higher error rate, possibly attributed to visual fatigue by radiologists. In this context, specific AI tools developed for lung cancer screening can automatically detect and measure lung nodules [53]. Lancaster et al. [54] recently assessed a type of deep learning tool, finding a reduction in negative misclassification errors compared to four out of five experienced radiologists. In another study, Jacobs et al. [55] demonstrated that using a dedicated viewer with automatic segmentation increased interobserver agreement while also reducing reading time.

Reducing reading time would help alleviate visual fatigue, resulting in a decrease in associated errors [55]. Particularly on breast cancer screening, there are numerous studies demonstrating how the application of AI algorithms can increase accuracy in the diagnosis of breast cancer [56]. For example, it has been demonstrated that the use of a setup with single-view digital breast tomosynthesis (DBT) and AI could enhance accuracy in cancer detection compared to the use of single-view DBT alone [57].

A study conducted in Sweden in 2023 demonstrated how AI can help increase breast cancer detection by 4% compared to double reading by two radiologists [58].

Also, interventional radiology can benefit most from AI, both in improving image processing and in guiding and predicting the outcomes of minimally invasive procedures. In particular, D’amore et al. [59] showcased how DL algorithms can optimize the probe’s trajectory during tumor ablation, minimizing damage to nearby structures. Meanwhile, Gurgitano et al. [60] illustrated how merging 3D anatomical data onto 2D fluoroscopic images enhances accuracy in pinpointing potential bleedings. These instances exemplify how AI can markedly diminish complications during procedures (type 11 error).

## 7. Regulatory and Ethical Considerations

The integration of AI and machine learning in radiology gives rise to numerous ethical and regulatory considerations concerning errors [61].

Firstly, patients should be informed about the use of AI in their diagnosis and treatment; therefore, informed consent becomes crucial in ensuring transparency regarding limitations and errors associated with AI systems [61].

To be trained properly, AI-based algorithms demand extensive datasets, necessitating the regulation of aspects related to their utilization and, overall, the data’s accuracy [62]. If data selection is not conducted meticulously, there is a potential for encountering the “GIGO” (garbage in–garbage out) phenomenon. This concept emphasizes that inaccurate, nonsensical, or incorrectly labeled input data can result in a nonsensical output. For this reason, it is crucial to ensure the integrity and accuracy of the information during training [63].

Ensuring fairness in the utilization of AI also involves addressing ethical concerns related to biases in AI algorithms [64,65].

For instance, an AI system with biases related to factors like race, gender, or socioeconomic status might result in disparities in diagnosis and treatment [64].

Another critical aspect is establishing liability in the case of an error associated with AI [62].

Currently, doctors can be legally responsible for poor patient outcomes if they fail to provide the appropriate standard of care. The future raises questions about who is responsible for adverse outcomes involving AI: whether it is the doctor using the AI tool, the software developer, or the purchasing hospital [62]. If a radiologist neglects to follow AI advice in the future, resulting in a negative patient outcome, overriding AI guidance could potentially be deemed as negligence by a court [62]. The recent AI Act has outlined rules and standards for the development, distribution, and oversight of AI systems in the European Union, categorizing them into four risk levels based on their potential impact on human rights, safety, and fundamental values. AI systems related to healthcare are considered high-risk and must adhere to strict rules regarding data quality, transparency, human oversight, accuracy, robustness, and security.

Furthermore, these systems must undergo a conformity assessment before being introduced to the market [66].

In the future, it is crucial to develop explicit guidelines for approving and consistently monitoring AI systems, ensuring their safety and effectiveness [61].

## 8. Barriers to Using AI in Radiology

In addition to regulatory and ethical considerations, there are several barriers to the widespread adoption of AI in radiology. One significant barrier is the fear of the unknown. Many radiologists and healthcare professionals may be apprehensive about relying on AI systems due to concerns about their reliability, accuracy, and the potential for unforeseen consequences [67]. This apprehension—those doctors and patients seem to share—is often rooted in a lack of familiarity with AI technologies and a fear that AI might replace human expertise rather than augment it [67].

Cost is another major barrier: the development, implementation, and maintenance of AI systems can be prohibitively expensive. High upfront costs for acquiring advanced AI technologies, coupled with ongoing expenses for software updates, training, and integration into existing healthcare infrastructures, can be a significant deterrent for many healthcare institutions, particularly those with limited budgets [68].

Additionally, the integration of AI in radiology requires substantial changes to existing workflows: radiologists need to adapt to new tools and processes, which can be time-consuming and disruptive [65]. There is also the challenge of ensuring that all staff are adequately trained to use these new systems effectively, which adds to the overall cost and complexity of implementation.

Data privacy and security concerns also pose significant barriers. The use of large datasets for training AI systems necessitates stringent measures to protect patient information. Ensuring compliance with data protection regulations, such as GDPR in the European Union, adds another layer of complexity to the deployment of AI technologies [69].

Moreover, there is a risk of bias in AI algorithms, which can lead to disparities in healthcare outcomes. If AI systems are trained on datasets that are not representative of the diverse patient population, they may not perform equally well for all demographic groups. This potential for bias requires careful consideration and ongoing monitoring to ensure the fair and equitable use of AI in radiology [70].

Lastly, there is the issue of liability. Establishing clear guidelines for accountability in cases where AI-assisted decisions lead to adverse patient outcomes is crucial. As AI systems become more integrated into clinical practice, determining the legal responsibility for errors—whether it lies with the radiologist, the AI developer, or the healthcare institution—becomes increasingly important [69].

## 9. Preventive Strategies

Learning from mistakes is considered an intrinsic part of human nature, contributing to the improvement in one’s skills and professional growth.

Examining the most common errors made in practice by radiologists and identifying their underlying causes is the first step in devising preventive strategies.

Among the causes of type 5 error there are inefficient team interaction, interprofessional tension, and communication failures [22].

As early as 2013, the ESR outlined guidelines aimed at promoting communication and interaction between radiologists and other physicians [21].

In the latter case, promoting collaboration between clinicians and radiologists is fundamental to preventing type 8 errors, linked to a lack of proper clinical context [22].

An innovative purpose is described in the study by Taussig et al. [71], whose intention was to setup a series of sessions during which fourth-year residents presented interpretative errors they had selected and offered relevant advice to junior residents [71]. This model, based on the creation of a judgment-free space, represents an opportunity to expand one’s knowledge, thoroughly exploring the nature of the error and discussing preventive measures with a peer audience to avoid the repetition of the same errors [71]. One could establish a weekly educational conference in the different radiology departments, creating a series of cases to be presented and discussed among the various residents. This could be an opportunity to share practical experiences to minimize “perceptual errors” and, more broadly, type 3 errors, commonly referred to as “lack of knowledge.” Incorporating root cause analysis into the training sessions for residents would be highly beneficial. This approach allows residents to recognize and address underlying issues more effectively, enabling them to proactively tackle potential problems. Weekly sessions would involve the selection and presentation of noteworthy cases, with a specific emphasis on those highlighting potential interpretation errors. Residents would have the opportunity to present cases and engage in in-depth discussions under the guidance of faculty. These sessions serve as genuine “update sessions,” fostering active case discussions that support ongoing learning and the prevention of future errors. This underscores the significance of collaboration and teamwork, playing a pivotal role in substantially mitigating errors.

In recent years, Scherer et al. [22] have highlighted the importance of multidisciplinary simulations in improving team coordination, but in the future, it is necessary to emphasize research on multidisciplinary team training and its direct effects on patient outcomes.

Certainly, the integration of AI would bring significant added value to radiological practice in terms of improved diagnostic practice and operational benefits [72].

Among these, it is worth emphasizing the diminishing of workloads and time savings, both crucial in mitigating the risk of burnout and psychophysical fatigue, an underestimated source of error.

Up until now, the majority of efforts have focused on intensive education for radiologists in training and retraining for practicing radiologists through continuous education [12]. Bruno et al. suggested approaches to minimize errors in the field of radiology, focusing particularly on ways radiologists could enhance their practical performance. However, the suggested measures, such as reducing work hours, alleviating the pressure to maintain a speedy workflow, and minimizing interruptions and distractions, ultimately had minimal impact. Accordingly, it could be innovative to conduct systematic studies to assess the effects of age or illnesses on a radiologist’s performance or to evaluate whether introducing routine visual acuity tests could lead to benefits, assuming that a decline in visual acuity might increase the risk of errors [12].
